# The Contribution of 17beta-Hydroxysteroid Dehydrogenase Type 1 to the Estradiol-Estrone Ratio in Estrogen-Sensitive Breast Cancer Cells

**DOI:** 10.1371/journal.pone.0029835

**Published:** 2012-01-09

**Authors:** Chen-Yan Zhang, Jiong Chen, Da-Chuan Yin, Sheng-Xiang Lin

**Affiliations:** 1 Laboratory of Molecular Endocrinology and Oncology, Centre Hospitalier de Université Laval Research Center (CHUL, CHUQ) and Laval University, Québec City, Québec, Canada; 2 Key Laboratory for Space Bioscience and Biotechnology, Faculty of Life Sciences, Northwestern Polytechnic University, Xi'an, Shaanxi, China; 3 Laboratory of Structural Biology with Visiting Scientists, Institute of Biochemistry and Cell Biology, Shanghai Institutes of Biological Sciences, Shanghai, China; 4 WHO Collaborative Center in Human Reproductive Research, Shanghai, China; Oklahoma Medical Research Foundation, United States of America

## Abstract

Estrone and estradiol are both estrogens with estrone being the less potent form and estradiol being the most potent estrogen. The binding of the latter to cellular regulatory elements stimulates the proliferation of breast cancer cells. A high ratio of estradiol/estrone is related to increased cell proliferation, and is of great importance to understanding of breast cancer mechanisms. 17beta-hydroxysteroid dehydrogenase type 1 and type 2 play important roles in the activation of estrone and inactivation of estradiol. Breast cancer cells T47D, MCF-7, BT 20, and JEG 3 as control cells, were chosen to evaluate the contribution of these two enzymes to the ratio. Twenty four hours after addition of different concentrations of estrone and estradiol, the ratio stabilized to around 9/1 in breast cancer cell lines with high expression of type 1 (T47D, BT 20, and JEG 3), whereas it approached 1/5 in cells with low expression of type 1 (MCF-7). The estradiol/estrone concentration ratio was modified to 9/1 in MCF-7 and HEK-293 cells over-expressing type 1. In T47D and BT 20, this ratio was decreased from 9/1 to nearly 1/5 (19/81 and 17/83 respectively) after type 1 knockdown by specific siRNAs. Type 2 is mainly involved in the conversion of estradiol into estrone. This ratio was decreased from 9/1 to 7/3 after over-expression of type 2 in MCF-7 cells already over-expressing type 1. The ratio was further decreased by the addition of the oxidative cofactor, NAD, to the cell culture to facilitate the estradiol to estrone conversion catalyzed by type 2. These results demonstrate that the estradiol/estrone ratio is controlled by both type 1 and type 2 with an additional contribution by NAD, although type 1 is the first determining factor in the cellular environment compared with type 2 and cofactors. Moreover, kinetic studies were carried out in intact cells as a new approach, using HEK-293 cells over-expressing type 1 and T47D breast cancer cells.

## Introduction

Breast cancer is one of the major causes of death in western women, with a 10% risk of survival [1]. In 2009, new breast cancer cases (192,370) were considerably more common than new lung cancer cases in US women (103,350) [2], [3]. Most breast cancer cases are estrogen dependent. Both the potent estrogen, estradiol (E2), and the less potent estrone (E1) are present in cancer cells [4]. E2 binds to estrogen receptors (ERα and ERβ) or to the G protein-coupled membrane receptor (GPR30), then recruits promoters of several genes related to proliferation, thus stimulating cell growth [1], [5], [6]. The high ratio of [E2]/[E1] (hereafter simplified as the ratio) in the cellular environment contributes significantly to BC cell proliferation [7]. In addition, the intratumoral [E2]/[E1] ratio was found to be significantly higher in postmenopausal women with a higher risk of breast cancer than in premenopausal women [8], [9], so decreasing this ratio (decreasing the production of E2) could be critically important for the therapy of breast cancer.

17beta-hydroxysteroid dehydrogenases (17β-HSDs) are important for the last step of estrogen and androgen activation and the first step of their degradation. E1 can be converted to E2 by reductive 17β-HSDs, and E2 can be converted to E1 by oxidative 17β-HSDs [10]–[13]. The highly active 17β-HSD1 plays an important role in E2 synthesis using NADPH as cofactor, with a Km value of 0.03±0.01 µM [14]. 17β-HSD2 plays an important role in E1 production using NAD as cofactor, with a Km value of 0.35±0.09 µM [15]. In some estrogen-dependent breast cancer cells, 17β-HSD1 is more abundantly expressed than 17β-HSD2 [16]–[18]. Using the purified protein it has been shown that 17β-HSD2 catalyzes E2 inactivation at a somewhat lower specific activity than that of E1 activation by 17β-HSD1, but it demonstrates much higher oxidative activity when compared to the other enzymes in the family known to date [19].

It has been reported that 17β-HSD1, 17β-HSD2 and the cofactor play important roles in the [E2]/[E1] ratio in HEK-293 cells over expressing the respective genes. The ratio of 92/8 in HEK-293 cells overexpressing 17β-HSD1 (HEK-293-17β-HSD1) was changed to 5/95 with overexpression of 17β-HSD2 [20]. Cofactors are also important for the conversion of estrogens. Recently, it has been demonstrated that this ratio was modified to 1/9 after mutagenesis of cofactor binding site R38 in HEK-293-17β-HSD1 [16]. The conversion rate of E2 to E1 was not changed significantly, after variation of the cofactor binding site in HEK-293-17β-HSD2 [16]. The evaluation of their importance to the [E2]/[E1] ratio in breast cancer cells (BC cells) requires further study in order to understand the mechanism of these estrogen dependence BC cells.

In this study, the contributions of 17β-HSD1, 17β-HSD2 and cofactor to the [E2]/[E1] ratio have been investigated directly from experiments in BC cells, as metabolite concentration ratios as proxies for metabolomics are becoming more common [21]. Our approach involved knocking down of 17β-HSD1, and simultaneous over-expression of 17β-HSD1 and 17β-HSD2 in BC cells to evaluate their contribution to this ratio. The results demonstrate that the expression of 17β-HSD1, is critically important for determining the [E2]/[E1] ratio in BC cells.

## Results

### 1. Contribution of 17β-HSD1 to [E2]/[E1] ratio in breast cancer cells

**Figure 1:**
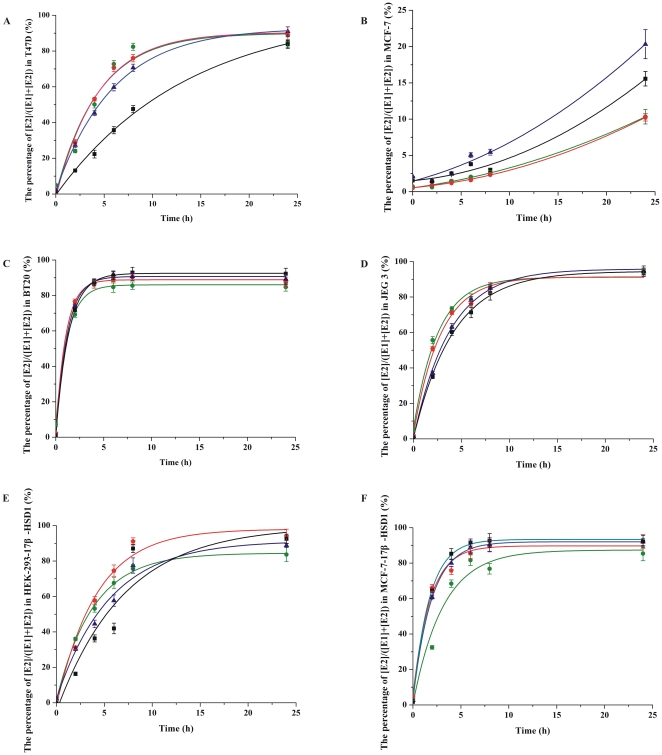
The conversion of E1 to E2 in T47D, MCF-7, BT 20, JEG 3, HEK-293-17β-HSD1 and MCF-7-17β-HSD1 cells. 50,000 cells were seeded onto 24-well plates, and different concentrations of E1 (0.01 µM, 0.05 µM, 0.1 µM and 0.3 µM) were added to dextran-coated charcoal treated medium. E2 production was evaluated every 0 h, 2 h, 4 h, 6 h, 8 h and 24 h. Choosing 0.01 µM, 0.05 µM, 0.1 µM and 0.3 µM E1 as the substrate are represented by green, red, blue and black lines respectively. Experiments were performed in duplicate, and all the data are fit to sigmoidal or exponential distribution (S.D.≤1%).

**Figure 2:**
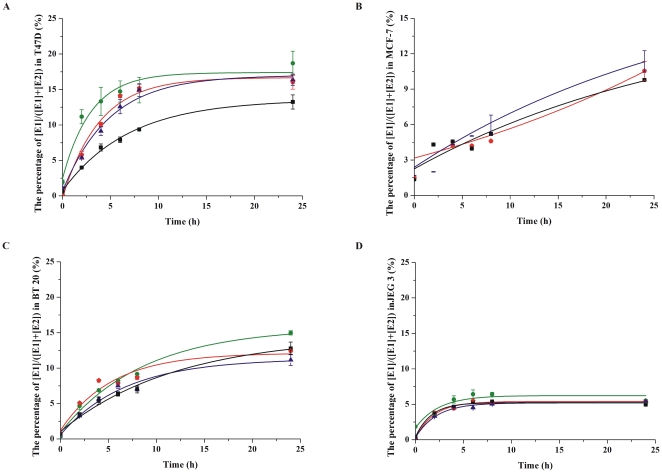
The conversion of E2 to E1 in T47D, MCF-7, BT 20 and JEG 3 cells. 50,000 cells were plated onto 24-well plates, and different concentrations of E2 (0.01 µM, 0.05 µM, 0.1 µM and 0.3 µM) were added to dextran-coated charcoal treated medium. E1 production was evaluated at 0 h, 2 h, 4 h, 6 h, 8 h and 24 h. Choosing 0.01 µM, 0.05 µM, 0.1 µM and 0.3 µM E2 as the substrate are represented by green, red, blue and black lines respectively. Experiments were performed in duplicate, and all the data are fit to sigmoidal or exponential distribution (S.D.≤1%).

The [E2]/[E1] ratio was analyzed in several cell lines after being cultured in presence of different concentrations of E1 and E2 for 24 hours. ER positive cell lines T47D and MCF-7, two other cell lines expressing 17β-HSD1 including an ER negative cell line BT 20, and a choriocarcinoma cell line JEG 3 as a control were used. Four different concentrations of E1 and E2 (0.01 µM, 0.05 µM, 0.1 µM and 0.3 µM) were added to the medium. The ratio was around 9/1 after incubation with E1 or E2 for 24 hours in all these chosen cell lines with high expression of 17β-HSD1 (including T47D, BT 20 and JEG 3) [17], [22], whereas it was less than 1/5 in MCF-7 cells which have a low expression of 17β-HSD1 (figure 1 and figure 2). The ratio stabilized after incubating different concentrations of E1 and E2 for 24, 48 and 72 hours, indicating that the system was in dynamic equilibrium.

Our results demonstrate that the ratio is maintained (around 9/1) in cell lines with high expression levels of 17β-HSD1, regardless of the concentration or steroids added to the culture medium. Proliferation of T47D and MCF-7 cells was evaluated after treatment with four different concentrations of E1. There was no significant increase of cell numbers after 24 hours, demonstrating that there is no correlation between this ratio and cell proliferation in the period when the ratio is evaluated.

**Figure 3:**
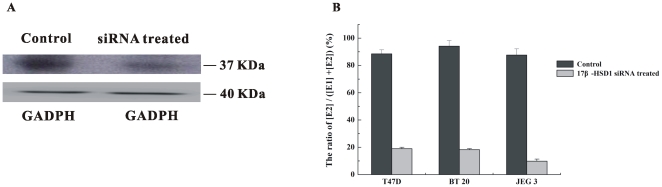
The conversion from E1 to E2 in T47D, BT 20 and JEG 3 cells after knocking down 17β-HSD1. A. 17β-HSD1 expression levels after being treated with specific siRNAs and control siRNAs (mixed SNC1 siRNA and scramble siRNA) in T47D cells. 200,000 cells were seeded onto 6-well plates, the cells were transfected with 100 nM mixed specific siRNAs for 17β-HSD1 or control siRNAs for 48 hours, then western blot was carried out. GADPH was chosen as the internal control protein. B. The conversion from E1 to E2 in T47D, BT 20 and JEG 3 cells after being treated with 17β-HSD1 specific siRNAs.

The ratio was decreased from 9/1 to 19/81 in T47D cells after treatment with 17β-HSD1 specific siRNAs, and was decreased from 9/1 to 17/83 in BT 20 cells after knocking down of 17β-HSD1 (figure 3). The ratio was increased to 9/1 after over-expression of 17β-HSD1 in HEK-293 cells and was increased from 1/5 to 9/1 after over-expression of 17β-HSD1 in MCF-7 cells (MCF-7-17β-HSD1). The expression level of 17β-HSD1 is thus a determining factor for the high ratio of [E2]/[E1] in BC cells.

### 2. The contribution of 17β-HSD2 to [E2]/[E1] ratio

**Figure 4:**
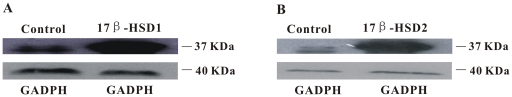
Western blots of 17β-HSD1 (A) and 17β-HSD2 (B) with over expressing in MCF-7. 100,000 cells were seeded onto 6-well plates, the cells were transfected with plasmid with over expressing of 17β-HSD1 and 17β-HSD2, then western blot was carried out after 48 hours. GADPH was chosen as the internal control protein.

In addition to 17β-HSD1, 17β-HSD2 also plays an important role in estrogen conversion, particularly for conversion of estradiol to estrone. MCF-7 cells were chosen to test the contribution of 17β-HSD1 and 17β-HSD2 to this ratio. MCF-7 cells over-expressing 17β-HSD1, those over-expressing 17β-HSD 2, and those over-expressing both 17β-HSD1 and 17β-HSD2 (MCF-7-17β-HSD-1&2) were constructed to evaluate this ratio (figure 4). The ratio was 1/9 in MCF-7-17β-HSD2. It was 9/1 in MCF-7-17β-HSD1, and decreased to 7/3 in MCF-7-17β-HSD1 after over expression of 17β-HSD2.

**Figure 5:**
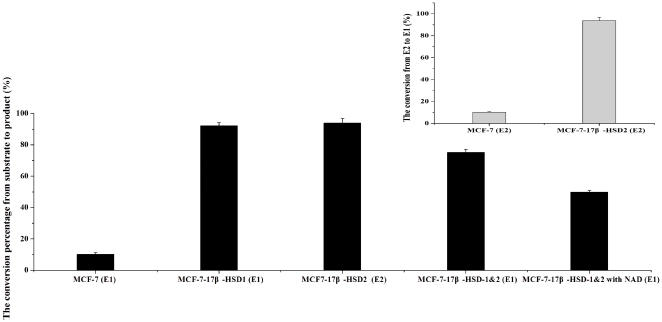
The conversion from substrate to product in MCF-7, MCF-7-17β-HSD1, MCF-7-17β-HSD1&2, MCF-7-17β-HSD1&2 with adding 0.5 mM NAD. Except MCF-7-17β-HSD2 using E2 as the substrate, E1 was chosen as the substrate in MCF-7, MCF-7-17β-HSD1 and MCF-7-17β-HSD1&2.

Cofactors also influence this ratio. E1 can be activated by 17β-HSD1 using the reductive cofactor NADPH, and E2 can be inactivated by 17β-HSD2 using the oxidative cofactor NAD. After addition of 0.5 mM NAD to the culture medium of MCF-7-17β-HSD-1&2, the conversion of E1 to E2 was decreased from 75.1% to 49.8%, and the conversion of E2 to E1 was increased from 27.2% to 57.6% (figure 5), both of them due to the increased type 2 oxidation. The ratio was unchanged by the addition of 0.5 mM NAD to the medium of T47D and MCF-7-17β-HSD1 cells. Thus, a decrease in [E2]/[E1] ratio is not caused by facilitating the oxidative reaction catalyzed by 17β-HSD1. The ratio is not affected by the addition of NAD in these two cell lines due to the fact that only tri-phosphate cofactors are specific for type 1 enzyme, as demonstrated by the enzyme structure and kinetic studies [14], [23]. Cell proliferation was investigated by the addition of 0.5 mM NAD to MCF-7-17β-HSD-1&2 cells. The decrease in [E2]/[E1] ratio was not a result of cell proliferation decrease after treatment of these cells with NAD. This demonstrates that 17β-HSD2 oxidation can be facilitated by increasing the NAD concentration and shows that the ratio is determined by the expression levels of 17β-HSD1, 17β-HSD2 and cofactors. Both of them are major determinants of this ratio in breast cancer cells while 17β-HSD1 comes for the first.

### 3. The enzyme kinetics of 17β-HSD1 activation of estrone in intact cells

**Figure 6:**
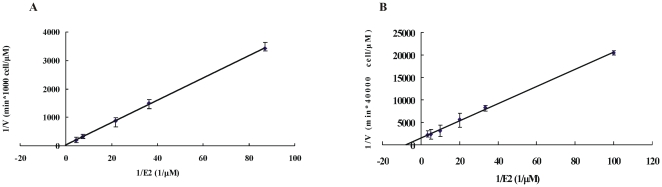
Double-reciprocal plot of 17β-HSD1 catalyzing estrone reduction in HEK-293-17β-HSD1 (A) and T47D (B). 1,000 HEK-293-17β-HSD1 cells and 40,000 T47D cells were seeded onto 24-well plates. E1 concentrations varied from 0.01 µM to 0.3 µM. Initial velocities were measured with less than 10% substrate consumption. The final concentration of ethanol was standardized to 0.05% in all reactions.

17β-HSD1 is important during the activation of estrogen. The enzyme kinetics have been studied with purified enzyme [14], [23], but not in a cellular context. HEK-293-17β-HSD1 and T47D cells (in which 17β-HSD1 dominates the conversion of E1 to E2) were chosen to study the steady-state kinetics of E1 activation in a cellular system. Different concentrations of E1, varying from 0.01 µM to 0.3 µM, were added to the medium. The apparent Km values were 0.25±0.04 µM and 0.12±0.02 µM for E1 to E2 conversion in HEK-293-17β-HSD1 and T47D respectively (figure 6).

## Discussion

A high level of E2 is positively correlated to breast cancer risk [24], [25], as this potent estrogen plays an important role in the proliferation of cancer cells [26]. It has been confirmed by microarray analysis that some genes related to cell growth and differentiation are regulated by E2, including PR (progesterone receptor), EREs (estrogen response elements), pS2 and cathepsin D [27], [28]. So a high [E2]/[E1] ratio in the cellular environment is closely related to the proliferation of BC cells and decreasing this ratio thus gives some insight into the therapy of breast cancer [4].

17β-HSDs are considered to be the main enzymes involved in the conversion of estrogens or androgens [5], [29], [30]. It has been proposed that 17β-HSD1 and 17β-HSD2 have important roles in determining the [E2]/[E1] ratio in HEK-293 where they are overexpressed [20]. Ratios of 9/1 in HEK-293-17β-HSD1, and 1/9 in HEK-293-17β-HSD2 have been reported [20]. In this study the [E2]/[E1] ratio was tested in BC cells and was found to be 9/1 in both ER positive and negative cells with a higher expression of 17β-HSD1. The ratio was decreased after knocking down the expression of 17β-HSD1 both in ER-positive cells and ER-negative cells, and it was increased from 1/5 in MCF-7 cells to 9/1 in MCF-7-17β-HSD1 cells. This demonstrates that the high ratio is positively correlated with 17β-HSD1 expression, while not correlative with the expression of estrogen receptors. No significant changes have been found for E1 to E2 conversion after knocking down other 17β-HSD reductive enzymes (data was not published), suggesting that 17β-HSD1 is a dominant factor in the maintenance of this ratio compared to other 17β-HSDs in breast cancer cells.

17β-HSD2 is considered to be an important enzyme for estrogen oxidation. The [E2]/[E1] ratio was decreased from 9/1 to 7/3 after over expression of 17β-HSD2 in MCF-7-17β-HSD1. This is consistent with the previous report that this ratio was slightly decreased after over expression of 17β-HSD2 in T47D cells [17]. Hence both 17β-HSD1 and 17β-HSD2 contribute to [E2]/[E1], whereas 17β-HSD1 is of the first importance in the cellular environment, consistent with its high activity.

The choice of cofactor is another important factor in the conversion of E1 to E2. The conversion of E2 to E1 can also be catalyzed by 17β-HSD1 with lower activity in vitro. During this oxidative conversion, the Kcat/Km value using NADP is 9.75 times greater than that obtained using NAD, so NADP is preferred during the oxidative conversion catalyzed by 17β-HSD1 [14]. [E2]/[E1] ratio was unchanged by the addition of 0.5 mM NAD to T47D and MCF-7-17β-HSD1 cells (with higher expression of 17β-HSD1 and lower expression of 17β-HSD2), whereas it was decreased by the addition of 0.5 mM NADP. The Kcat/Km value for 17β-HSD2 using NAD is 400 times greater than that using NADP, so NAD is the preferred cofactor for its major role in E2 oxidation [15]. In addition, the specificity of 17β-HSD2 to NAD is around 3 times higher than that of 17β-HSD1 [14]–[16]. [E2]/[E1] was further decreased from 7/3 to 1/1 by the addition of 0.5 mM NAD to MCF-7-17β-HSD-1&2 cells, but it was not significantly changed by the addition of 0.5 mM NAD to MCF-7-17β-HSD-1 cells. The decrease in this ratio in MCF-7-17β-HSD-1&2 cells by addition of NAD is due to facilitation of the oxidation catalyzed by 17β-HSD2. Furthermore it has been confirmed that the decrease in [E2]/[E1] ratio after addition of 0.5 mM NAD as the cofactor is not due to cell death. In the cellular environment, 17β-HSD1 reduction is predominant over its oxidation to yield the high [E2]/[E1] ratio, for which 17β-HSD1 is of the first importance as compared to 17β-HSD2 and cofactor in BC cells.

17β-HSD1 kinetics have been studied using purified enzyme [14], [23]. The apparent affinity is different for different cofactors such as NADP, NADPH, NAD and NADH. NADPH has the highest specificity for the conversion of estrogens. The Km value of estrone activation by purified 17β-HSD1 using NADPH is 0.03±0.01 µM. Until now, the Km value of 17β-HSD1 in the cellular environment has not been extensively studied. HEK-293-17β-HSD1 and T47D were chosen to measure the Km value in intact cells. Although other reductive 17β-HSDs enzymes (17β-HSD5, 17β-HSD7 and 17β-HSD12) can convert E1 to E2 in T47D, it has been shown that 17β-HSD1 is the dominant enzyme for this catalysis in the cell line [18], [31]. This demonstrates the high specificity of 17β-HSD1 for the estrone substrate. The apparent Km value for 17β-HSD1 is 4–8 times larger in the cellular environment compared to the purified enzyme.

### Conclusion

A high [E2]/[E1] ratio is positively correlated to the proliferation of BC cells [7], and its reduction is suggested to be an effective means of facilitating breast cancer therapy. This ratio was around 9/1 in BC cells with a high expression level of 17β-HSD1. The ratio was significantly increased in MCF-7-17β-HSD1 compared to MCF-7 cells, but was decreased after knocking down 17β-HSD1 in T47D and BT 20 cells. The ratio decreased with the additional expression of 17β-HSD2 in MCF-7-17β-HSD1, and further decreased with adding oxidative cofactor NAD in the culture medium. 17β-HSD1 remains the first determinant of the high ratio in BC cells when compared to17β-HSD2 and cofactors. Furthermore, the kinetic studies were carried out in HEK-293-17β-HSD1 and T47D cells, demonstrating the high apparent specificity of this enzyme to the estrone substrate.

## Materials and Methods

### Chemicals

The E1 and E2 radiochemicals were obtained from American Radiolabeled Lab (St. Louis, MO), and unlabeled steroids were purchased from Sigma (Oakville, Ontario, Canada). All steroids were dissolved in ethanol. NAD cofactor was purchased from Sigma.

### Cell culture

ER positive cell lines T47D and MCF-7, ER negative cell line BT 20, HEK-293 cells, and the choriocarcinoma cell line JEG 3 were obtained from American Type Culture Collection (ATCC, Manassas, VA). MCF-7 cells were maintained in DME low-glucose medium. T47D cells were propagated in DME high-glucose medium containing 7.5 mg/L bovine insulin (Sigma, Oakville, Ontario, Canada). Phenol red-free medium was used. BT 20, JEG 3 and HEK-293 cells were cultured in MEM medium. All the media were supplemented with 10% FBS, and cultured in an incubator at 37 C under 5% CO_2_ humidified atmosphere.

### siRNA synthesis, plasmid construction and transfection

17β-HSD1 siRNA was designed according to the previous study [7]. siRNA transfections were carried out by lipofectamine 2000 (Invitrogen, Burlington, Ontario, Canada), following the manufacturer's instructions. 40,000–50,000 cells were seeded onto the 24-well plates one day before transfection, and 100–200 nM mixed siRNA duplexes were transfected. Silencer negative control siRNA (Ambion, Austin, TX) and scramble siRNA (GenePharma, Shanghai, China) were used as control siRNAs. Three pairs of siRNAs were synthesized for 17β-HSD1, including siRNA 1 (5′- GCUGGACGUGAAUGUAGUATT-3′, 5′- UACUACAUUCACGUCCAGCTT-3′), siRNA 2 (5′- GCCUUUCAAUGACGUUUAUTT-3′, 5′- AUAAACGUCAUUGAAAGGCTT-3′) and siRNA 3 (5′- CCACAGCAAGCAAGUCUUUTT-3′, 5′-AAAGACUUGCUUGCUGUGGTT-3′) [7].

Human 17β-HSD1 and 17β-HSD2 cDNAs were amplified using specific primers, and the fragments were inserted into the pcDNA3.1 (+) vector. Recombinant plasmids 17β-HSD1 and 17β-HSD2 were transfected into cells with lipofectamine 2000. 200,000 MCF-7 cells over-expressing 17β-HSD1 and 17β-HSD2 were seeded onto 6 well-plates. All the enzyme activity analyses were carried out after being treated with siRNAs and recombinant plasmid for 48 hours.

### Western blot

Total protein was extracted from cells lysed with RIPA buffer supplemented with 1 mM Phenylmethanesulfonyl fluoride and a protein inhibitor cocktail (Santa Cruz Biotechnology, Santa Cruz, CA). Equal amounts of total protein were loaded onto a 12% SDS polyacrylamide gel and transferred onto a nitrocellulose membrane (PerkinElmer, Waltham, MA). The membrane was blocked with 5% nonfat milk in PBS-Tween buffer overnight. The membrane was further incubated with PBS-Tween buffer containing a 1∶ 100,000 dilution of the primary anti-17β-HSD1 rabbit polyclonal antibody (Abcam, Cambridge, MA) or 1∶ 1,500 dilution of primary anti-17β-HSD2 mouse polyclonal antibody (Abcam, Cambridge, MA). The respective horseradish peroxidase-conjugated antibody (Santa Cruz Biotechnology) was chosen as the secondary antibody and was diluted by a factor of 6,000. Protein signals were visualized with chemiluminescence reagent (PerkinElmer).

### Activity assay in several cell lines

50,000 cells were plated onto 24-well plates for testing enzyme activity. Different concentrations of [14C] E1 and [14C] E2 (0.01 µM, 0.05 µM, 0.1 µM and 0.3 µM) were added to the medium treated with dextran-coated charcoal. The cell supernatants were collected after incubation at 37 C with 5% CO_2_ for the indicated times. The final content of ethanol was standardized to 0.05%.

Kinetics experiments for cells with overexpression or knock down of respective genes were carried out after transfection with 100 nM siRNAs and recombinant plasmid for 48 hours. A final concentration of 0.05 µM [14C]-E1 was added to the culture medium and the cell supernatants were collected after incubation at 37 C with 5% CO_2_ for 24 hours.

When evaluating Km values in T47D and HEK-293-17β-HSD1 cells, the concentrations of E1 varied from 0.01 µM to 0.3 µM. Different numbers of cells were used for these two cell lines and initial velocities were measured with less than 10% of substrate consumption. E1 was added to the medium and the reaction was stopped at 0 min, 10 min, 20 min and 30 min.

After the incubation, the steroids were extracted with 3 volumes of diethyl ether on ethanol dry ice bath. The organic phase was evaporated, dissolved in 50 µl dichloromethane, applied to Silica gel 60 thin layer chromatography plates (Merck, Darmstad, Germany), and separated by migration in a toluene-acetone (4∶1, v/v) solvent system. TLC plates were exposed and quantified using a storm imaging system (Molecular Dynamics, Sunnyvale, CA).

### Cell proliferation analysis

Cell proliferation was determined by the 3-(4, 5-Dimethylthiazol-2-yl)-2, 5-diphenyltetrazolium bromide (MTT) assay. 50,000 cells were plated into each well of a 24-well plate, and different concentrations of E1 and E2 (0.01 µM, 0.05 µM, 0.1 µM and 0.3 µM) were added to the medium. MTT assays were carried out after 24 hours following the manufacturer's instructions. Absorbency was measured on a plate reader (Spectra Max 340PC, Molecular Devices, Sunnyvale, CA) at 570 nm, with 630 nm as a reference.

## References

[1] Lin SX, Chen J, Mazumdar M, Poirier D, Wang C (2010). Molecular therapy of breast cancer: progress and future directions.. Nat Rev Endocrinol.

[2] American Cancer Society. http://www.cancer.org/docroot/cri/content/cri_2_4_1x_what_are_the_key_Statistics_about_lung_cancer_15.

[3] American Cancer Society. http://www.cancer.org/downloads/STT/F861009_final%209-08-09.pdf.

[4] Suzuki T, Miki Y, Nakamura Y, Moriya T, Ito K (2005). Sex steroid-producing enzymes in human breast cancer.. Endocr Relat Cancer.

[5] Fox EM, Andrade J, Shupnik MA (2009). Novel actions of estrogen to promote proliferation: Integration of cytoplasmic and nuclear pathways.. Steroids.

[6] Gunnarsson C, Olsson BM, Stål O (2001). Abnormal expression of 17beta-hydroxysteroid dehydrogenases in breast cancer predicts late recurrence.. Cancer Res.

[7] Aka JA, Mazumdar M, Chen CQ, Poirier D, Lin SX (2010). 17β-hydroxysteroid dehydrogenase type 1 stimulates breast cancer by dihydrotestosterone inactivation in addition to estradiol production.. Mol Endocrinol.

[8] Miyoshi Y, Ando A, Shiba E, Taguchi T, Tamaki Y (2001). Involvement of up-regulation of 17β-hydroxysteroid dehydrogenase type 1 in maintenance of intratumoral high estradiol levels in postmenopausal breast cancers.. Int J Cancer.

[9] Jansson A, Delander L, Gunnarsson C, Fornander T, Skoog L (2009). Ratio of 17HSD1 to 17HSD2 protein expression predicts the outcome of tamoxifen treatment in postmenopausal breast cancer patients.. Clin Cancer Res.

[10] Meier M, Möller G, Adamski J (2009). Perspectives in understanding the role of human 17β-hydroxysteroid dehydrogenases in health and disease.. Ann N Y Acad Sci.

[11] Moeller G, Adamski J (2009). Integrated view on 17beta-hydroxysteroid dehydrogenases.. Mol Cell Endocrinol.

[12] Jansson A (2009). 17beta-hydroxysteroid dehydrogenase enzymes and breast cancer.. J Steroid Biochem Mol Biol.

[13] Peltoketo H, Luu-The V, Simard J, Adamski J (1999). 17beta-hydroxysteroid dehydrogenase (HSD)/17-ketosteroid reductase (KSR) family; nomenclature and main characteristics of the 17HSD/KSR enzymes.. J Mol Endocrinol.

[14] Jin JZ, Lin SX (1999). Human estrogenic 17beta-hydroxysteroid dehydrogenase: predominance of estrone reduction and its induction by NADPH.. Biochem Biophys Res Commun.

[15] Lu ML, Huang YW, Lin SX (2002). Purification, reconstitution, and steady-state kinetics of the trans-membrane 17beta -hydroxysteroid dehydrogenase 2.. J Biol Chem.

[16] Sherbet DP, Guryev OL, Papari-Zareei M, Mizrachi D, Rambally S (2009). Biochemical factors governing the steady-state estrone/estradiol ratios catalyzed by human 17beta-hydroxysteroid dehydrogenases types 1 and 2 in HEK-293 Cells.. Endocrinology.

[17] Miettinen MM, Mustonen MV, Poutanen MH, Isomaa VV, Vihko RK (1996). Human 17 beta-hydroxysteroid dehydrogenase type 1 and type 2 isoenzymes have opposite activities in cultured cells and characteristic cell- and tissue-specific expression.. Biochem J.

[18] Laplante Y, Rancourt C, Poirier D (2009). Relative involvement of three 17beta-hydroxysteroid dehydrogenases (types 1, 7 and 12) in the formation of estradiol in various breast cancer cell lines using selective inhibitors.. Mol Cell Endocrinol.

[19] Jansson A, Gunnarsson C, Stål O (2006). Proliferative responses to altered 17beta-hydroxysteroid dehydrogenase (17HSD) type 2 expression in human breast cancer cells are dependent on endogenous expression of 17HSD type 1 and the oestradiol receptors.. Endocr Relat Cancer.

[20] Khan N, Sharma KK, Andersson S, Auchus RJ (2004). Human 17beta-hydroxysteroid dehydrogenases types 1, 2, and 3 catalyze bi-directional equilibrium reactions, rather than unidirectional metabolism, in HEK-293 cells.. Arch Biochem Biophys.

[21] Illig T, Gieger C, Zhai GJ, Margl WR, Sattler RW (2010). A genome-wide perspective of genetic variation in human metabolism.. Nat Genet.

[22] Smuc T, Rizner TL (2009). Expression of 17beta-hydroxysteroid dehydrogenases and other estrogen-metabolizing enzymes in different cancer cell lines.. Chem Biol Interact.

[23] Gangloff A, Garneau A, Huang YW, Yang F, Lin SX (2001). Human oestrogenic 17beta-hydroxysteroid dehydrogenase specificity: enzyme regulation through an NADPH-dependent substrate inhibition towards the highly specific oestrone reduction.. Biochem J.

[24] Pasqualini JR, Chetrite G, Nguyen BL, Maloche C, Delalonde L (1995). Estrone sulfate-sulfatase and 17[beta]-hydroxysteroid dehydrogenase activities: a hypothesis for their role in the evolution of human breast cancer from hormone-dependence to hormone-independence.. J Steroid Biochem Mol Biol.

[25] Pasqualini JR, Chetrite G, Blacker C, Feinstein MC, Delalonde L (1996). Concentrations of estrone, estradiol, and estrone sulfate and evaluation of sulfatase and aromatase activities in pre- and postmenopausal breast cancer patients.. J Clin Endocrinol Metab.

[26] Castoria G, Migliaccio A, Giovannelli P, Auricchio F (2010). Cell proliferation regulated by estradiol receptor: Therapeutic implications.. Steroids.

[27] Laganière J, Deblois G, Lefebvre C, Bataille AR, Robert F (2005). From the cover: location analysis of estrogen receptor alpha target promoters reveals that FOXA1 defines a domain of the estrogen response.. Proc Natl Acad Sci USA.

[28] Cicatiello L, Addeo R, Sasso A, Altucci L, Petrizzi VB (2004). Estrogens and progesterone promote persistent CCND1 gene activation during G1 by inducing transcriptional derepression via c-Jun/c-Fos/estrogen receptor (progesterone receptor) complex assembly to a distal regulatory element and recruitment of Cyclin D1 to its own gene promoter.. Mol Cell Biol.

[29] Peltoketo H, Isomaa V, Mäentausta O, Vihko R (1988). Complete amino acid sequence of human placental 17beta-hydroxysteroid dehydrogenase deduced from cDNA.. FEBS Letters.

[30] Wu L, Einstein M, Geissler WM, Chan HK, Elliston KO (1993). Expression cloning and characterization of human 17 beta-hydroxysteroid dehydrogenase type 2, a microsomal enzyme possessing 20 alpha-hydroxysteroid dehydrogenase activity.. J Biol Chem.

[31] Aka JA, Mazumdar M, Lin SX (2009). Reductive 17beta-hydroxysteroid dehydrogenases in the sulfatase pathway: Critical in the cell proliferation of breast cancer.. Mol Cell Endocrinol.

